# Assessment of Initial Vancomycin Dosing in Pediatric Oncology Patients

**DOI:** 10.3390/children4090079

**Published:** 2017-08-28

**Authors:** Hillary Orr, Deni Trone, Joshua Elder, Ashok Raj

**Affiliations:** 1 Department of Pharmacy, Norton Children’s Hospital, Louisville, KY 40202, USA; Dtrone@stjude.org (D.T.); Joshua.Elder@nortonhealthcare.org (J.E.); 2 Department of Pharmacy, Children’s Memorial Hermann Hospital, 6411 Fannin Street, Houston, TX 77030, USA; 3 Department of Pharmacy, St. Jude Children’s Research Hospital, 262 Danny Thomas Place, Memphis, TN 38105, USA; 4 Division of Pediatric Hematology-Oncology, University of Louisville, Louisville, KY 40202, USA; Ashok.Raj@louisville.edu

**Keywords:** vancomycin, pediatrics, oncology, pharmacokinetics

## Abstract

This was a retrospective audit assessing vancomycin dosing of 60 mg/kg/day in the attainment of therapeutic concentrations between 10–20 mcg/mL among 56 pediatric oncology patients. Twelve patients (21%) achieved therapeutic concentrations of 10–20 mcg/mL, while 44 patients (79%) obtained trough levels below 10 mcg/mL despite the addition of nephrotoxic agents.

## 1. Introduction

Neutropenic fever is considered a medical emergency among oncology patients and needs to be treated with broad-spectrum antibiotics to decrease morbidity and mortality [1]. Gram-positive bacteria are the most frequently occurring causative microorganisms of fever, making up approximately 60% to 70% of documented infections in these patients [1,2,3]. Current consensus recommendations in pediatric oncology specify the clinical indications necessitating the use of vancomycin therapy: suspected catheter-related infection, skin and soft-tissue infections, pneumonia, severe mucositis, or hemodynamic instability [4].

The initiation of a dosing regimen that produces therapeutic vancomycin trough concentrations directly upon the start of therapy is crucial; however, there is limited data that exists regarding the optimal dosing in pediatric oncology patients [2,5,6]. Previous studies have documented that higher vancomycin dosing regimens may be required to achieve therapeutic concentrations in pediatric patients due to increased clearance, smaller volume of distribution, and shorter half-life [2,6]. Current guidelines recommend pediatric dosing of vancomycin 40–60 mg/kg/day, but literature reports that doses of 80–90 mg/kg/day may be required to attain therapeutic levels [1,2,3].

Although literature scarcely addresses the correlation of therapeutic vancomycin trough concentrations with improvements in mortality due to infection, there is evidence among the general population that suggests that patients with trough concentrations ≥10 mcg/mL during the presence of an infection were more likely to become afebrile [7]. Additionally, little is known regarding vancomycin dosing in oncology patients who have developed decreased renal function due to concomitant nephrotoxic agents and underlying malignancies.

The purpose of this medication use evaluation is to assess initial vancomycin dosing regimens and the resulting dose and interval required for the attainment of therapeutic concentrations of 10–20 mcg/mL in a pediatric oncology population receiving vancomycin 60 mg/kg/day.

## 2. Methods

This is a retrospective audit conducted at an academic teaching hospital involving pediatric oncology patients who were empirically initiated on vancomycin therapy from March 2013 to November 2015. Clinical indications for the addition of vancomycin in pediatric oncology patients include suspected catheter-related infection, skin and soft-tissue infections, pneumonia, severe mucositis or hemodynamic instability. Patients were initially administered vancomycin 60 mg/kg/day and trough levels were collected within 30 minutes of a scheduled dose, after a minimum of 3 doses, with clinical dosing support provided by a pediatric clinical pharmacist. Inclusion criteria were children between 6 months to 18 years of age with an oncologic diagnosis, vancomycin dosage of 60 mg/kg/day (±10%) divided every 6–8 h, and each patient must have received at least 3 doses of vancomycin prior to obtainment of a vancomycin trough level to ensure steady state concentration. Therapeutic trough levels were defined as 10–20 mcg/mL. Exclusion criteria were vancomycin trough levels drawn prior to steady state, a change in serum creatinine greater than 0.3 mg/dL from baseline, patients requiring renal replacement therapy and patients admitted to the pediatric intensive care unit. An extensive chart review was performed to gather the following: age, weight, underlying diagnosis, serum creatinine, urine output, indication for vancomycin, vancomycin doses, trough concentrations, and concomitant nephrotoxic agents, including intravascular injection of iodinated radiographic contrast media, methotrexate, and other chemotherapeutic agents, calcineurin inhibitors, or aminoglycoside antibiotics.

The information collected was evaluated for percentage of patients having a therapeutic trough with the initial vancomycin dosing regimen of 60 mg/kg/day. If initial dosing was evaluated to be subtherapeutic, dosing required to achieve therapeutic trough levels was evaluated. Initial dosing and corresponding first trough levels were then compared with those patients requiring dose adjustments to achieve therapeutic trough levels. Fisher exact test was utilized to compare age groups, corresponding trough levels with increased dosing regimens, and the presence of concomitant nephrotoxic agents.

## 3. Results

Initial dosing of vancomycin 60 mg/kg/day was prescribed for all patients in the study. Overall, 10 out of 48 patients achieved therapeutic trough concentrations between 10 and 20 mcg/mL with initial vancomycin dosing. Of the 10 patients who achieved therapeutic trough concentrations, 7 of these patients were 10 years of age or greater.

Six out of 28 leukemia patients (21%) achieved therapeutic trough concentrations with initial dosing. Neither of the 2 lymphoma patients demonstrated therapeutic vancomycin trough concentrations with initial dosing. Only one patient (14%) in the central nervous system (CNS) tumor group and 3 patients (27%) in the sarcomas group achieved therapeutic vancomycin concentrations. A comparison of patients younger than 10 years of age with those 10 years of age or older was evaluated due to the fact that these patients may have increased clearance of vancomycin. The probability of a subtherapeutic level was higher in patients younger than 10 years of age as compared to patients 10 years of age or older (*p* = 0.028). These findings are illustrated in Table 1.

After evaluating the corresponding trough levels recorded for the initial vancomycin dosing regimen, adjustments in the dose and/or interval were implemented to evaluate for the attainment of therapeutic vancomycin concentrations. Nineteen patients were recorded to have subtherapeutic vancomycin trough concentrations with the initial dosing regimen and required a dosage increase to 80 mg/kg/day. Of the 19 patients that were recorded to have a dosage increase with subsequent levels drawn, 12 patients (63%) achieved therapeutic levels. The remaining 7 patients persisted to have subtherapeutic concentrations despite a dosage increase to 80 mg/kg/day. Subsequently, these patients were increased to a dosing regimen of vancomycin 100 mg/kg/day, which resulted in therapeutic trough concentrations. The remaining 19 patients were unaccounted for due to the discontinuation of vancomycin therapy after clinical indications necessitating the continuation of therapy were no longer met and thus, negated the need for follow-up levels. Thirteen patients evaluated in the study had documented recent use of concomitant nephrotoxic medications including tacrolimus, aminoglycosides, radiographic contrast media, and chemotherapeutic agents (i.e., methotrexate, cisplatin). There was no incidence of renal dysfunction among patients included in the study prior to the initiation of vancomycin therapy. Of those with exposure to concomitant nephrotoxic agents, there was no significant association with observations of renal dysfunction or increased vancomycin concentrations (*p* = 0.108). Five of the 13 patients receiving concomitant nephrotoxic agents demonstrated therapeutic trough concentrations with vancomycin 60 mg/kg/day.

## 4. Conclusions

This study concludes that the majority of pediatric oncology patients initiated on vancomycin 60 mg/kg/day demonstrated subtherapeutic trough concentrations. Additionally, 90% of patients younger than 10 years of age experienced subtherapeutic vancomycin trough concentrations, which was a significant finding. This suggests that younger patients appear to require higher doses of vancomycin and demonstrate greater variability in vancomycin dosage requirements to achieve adequate vancomycin trough concentrations. A vancomycin dosing regimen of at least 80 mg/kg/day may be required to attain therapeutic levels in pediatric oncology patients despite the presence of additional nephrotoxic agents having the potential to reduce the clearance of vancomycin.

## Figures and Tables

**Table 1:** Patient and vancomycin pharmacokinetic characteristics.

Characteristics	Number of Subjects	<10 mcg/mL (%)	10–20 mcg/mL (%)	*p*
**Dosing Regimen**				
60 mg/kg/day	48	38 (79%)	10 (21%)	
**Subsequent Dosing Regimen**				
80 mg/kg/day	19	7 (37%)	12 (63%)	
100 mg/kg/day	7	0 (0%)	7 (100%)	
**Age**				
<10 year of age	30	27 (90%)	3 (10%)	0.03
≥10 year of age	18	11 (61%)	7 (39%)	
**Diagnosis**				
Leukemia	28	22 (79%)	6 (21%)	1.00
Lymphoma	2	2 (100%)	0 (0%)	1.00
CNS Tumors	7	6 (86%)	1 (14%)	1.00
Sarcomas	11	8 (73%)	3 (27%)	0.68
**Nephrotoxic Agents**				
Present	13	8 (62%)	5 (38%)	0.11
Not Present	35	30 (86%)	5 (14%)	

CNS: central nervous system.
